# Hospital and Health News

**Published:** 1924-04

**Authors:** 


### HOSPITAL AND HEALTH NEWS.

### A Trustee's Gift.

Chesterfield Royal Hospital has received a gift of £10,000 from Mr. Charles Markham, who is one of the hospital's trustees.

### General Nursing Council for Scotland.

The preliminary examination of the General Nursing Council for Scotland begins on April 15, on which the written paper will be taken simultaneously at the different centres.

### A School for Magistrates.

The Central Association for Mental Welfare is holding a course of instruction for magistrates in connection with their duties under the Lunacy and Mental Deficiency Acts.

### Another Orthopædic Clinic.

An orthopædic clinic for crippled children is proposed for Mansfield as part of a scheme for Nottingham and the county. A joint open-air country hospital is also contemplated.

### Small-pox Hospital for Devon.

The Devon Public Health Committee has secured a site four miles from Exeter for a county small-pox hospital. Plymouth is willing to co-operate in providing the necessary accommodation.

### Co-operative Scheme of Benefits.

Brixham Cottage Hospital has arranged a "co-operative scheme of benefits" with organised labour, which suggests that cottage hospitals also can adopt the so-called insurance plan of regular subscriptions.

### Found in a Collecting Box.

A cheque for threepence, duly honoured since, the return half of an Underground ticket, and 120 farthings in a flour-bag, have been contributed to the collecting-box at the West London Hospital.

### A Difficult Year.

The London Fever Hospital, which is not so well known as it ought to be, has passed a difficult year because of a relative absence of epidemic. Thus the fees received from patients were much reduced, and the institution is forced to draw upon its capital.

### Twenty Miles from a Hospital.

The Kinsale Board of Guardians, Ireland, has passed a resolution urging the need for a hospital at Kinsale, because, apart from immediate necessity, the sending of patients a distance of twenty miles to Cork District Hospital is inconvenient and sometimes impracticable.

### An Unsuccessful Appeal.

The Royal Bath Hospital and Rawson Convalescent Home received only £1,800 of the £12,000 asked for in its recent centenary appeal. It is now open winter as well as summer, and the record number of patients received has justified the change. A new laboratory is about to be established.

### Lectures at Strasbourg.

A practical course of dermatology and venereal disease is to be given at the Clinic for Cutaneous and Syphilitic Diseases, Strasbourg, from September 22 to November 8. The course is being organised in connection with the Faculty of Medicine, Strasbourg University, by Professor L. D. Pautrier.

### Mothercraft.

The National League for Health, Maternity and Child Welfare have just issued a fourth edition of their book, "Mothercraft." The new edition of this useful volume, which deals clearly and concisely with all matters relating to the education of mothers and the care of children, can be obtained for the moderate price of 4s. 6d.

### Blind Students' Success.

At the recent Electricity Examination for Blind Students four students of the Massage School of the National Institute for the Blind entered. Three of these qualified in the full examination and one passed in the practical examination. The students are prepared for examination by a blind instructor.

### A New "Hospital" for London.

The London Hospital for Refraction, it is announced, offers post-graduate training to qualified opticians, and also provides free attendance for poor people. Mr. Hartley Shaw, a Bradford optician, is one of the founders of the institution.

### Death of Mr. W. G. Deedes.

The death of Mr. W. G. Deedes, following so soon after his resignation of the hon-secretaryship of the Exmouth Cottage Hospital, is much regretted. He was an example of the silent but splendid work done by honorary workers for our cottage hospitals, and had held the secretaryship for over eleven years. A testimonial fund was being raised for him, which we hope will be completed for the benefit of his family, to whom public acknowledgment of Mr. Deedes's work is due.

### Fire at a Children's Hospital.

A fire recently broke out in the sewing-room at the Royal Alexandra Hospital for Children, Brighton, and was discovered by the night sister, Miss Wood. She gave the alarm and started work with the hose from the hospital hydrant. With the help of the fire brigade and the nurses the outbreak was mastered and restricted to the room in which it occurred, the central position of which at the top of the main flight of stairs rendered the risk considerable.

### Financial Condition of King's College Hospital.

The financial condition of King's College Hospital is an index to be watched with anxiety, but the past year shows that the income covered the expenditure upon the reduced number of wards, and even left a small balance toward the debt of £73,000, thanks to the festival dinner and the final grant of £7,000 from the combined Appeal. It is hoped that the coming Pageant at the Crystal Palace will make good these special and non-recurring sums.

### New Medical School at Khartoum.

Sir L. Stack, Governor-General of the Soudan, last month formally opened the Kitchener Medical School at Khartoum in the presence of leading members of the European and native communities. The trustees of the Gordon College have consented to act as trustees of the new medical school, which, situated adjacent to the Khartoum Civil Hospital, is a large, airy, graceful building, erected and equipped at a cost of £26,000, £13,000 of which has been collected in the Soudan, mostly from natives.

### Appeal Week for Westminster Hospital.

From April 7 to April 12 Westminster Hospital is to hold its special appeal week in order to raise £35,000, the remaining half of the sum required to remodel the building. A matinée and drawing-room meetings are to be held, and on June 17 a bazaar, at which pieces from the original stone of the Abbey will be on sale. These remain from unserviceable fragments revealed by the restoration. The Prince of Wales, the president of the hospital, will reopen it in July.

### The American Hospital in London.

The Provisional Committee of the American Hospital in London are now ready to receive patients at their temporary headquarters, Manor House, 47 St. John's Wood Park, N.W. 8. The patients are limited to American citizens, American wives of British subjects and their children. Applications for the admission of patients and all communications relative to the hospital should be directed to Mr. Philip Franklin, F.R.C.S., Medical Director, 27 Wimpole Street, W. 1.

### The Care of Infants.

A course of lectures to nurses, health visitors and others interested in maternity and child welfare or in the care of infants will be held at the Infants' Hospital, Vincent Square, S.W. 1, between April 2 and June 25, each Wednesday at 6 p.m. Tickets for the course are 5s. and for a single lecture 1s., and are obtainable from the secretary of the hospital.

### New Name for a Hospital.

The Sussex Maternity and Women's Hospital is the new name that was adopted for the Brighton and Hove hospital for women at the meeting held at the end of the first year's complete work in the Buckingham-road premises. Some spare rooms have been converted into paying wards, and show signs of being in constant request. The change of name is intended to indicate that the hospital serves more than local needs, and will also avoid confusion with the new Women's Hospital, from which both have suffered. The discarded name has been in use since 1830, when the hospital was founded.

### Baby Welfare.

Dr. Bullough, County Medical Officer for Essex, has revised his useful little pamphlet on "How to Take Care of Baby," which is published by the Essex County Council. Concise and practical, it is the very thing to put into the hands of working-class mothers. The list of Child Welfare Centres, with days and times of sessions, should be of special service to them. Another little book for mother is published by Messrs. Routledge, at the price of 6d. It is entitled "Hints to expectant Mothers and on the Care of an Infant," and its author is Miss Ada Underwood, C.D.B. This also may be thoroughly recommended.

### A Silent and Sealed Churn.

The clatter of milk-cans at railway stations is one of the annoyances to which, apparently, the public need no longer be exposed. A new type of railway milk-churn, fitted with rubber tyres, has been designed by Mr. A. J. F. Robinson, of Barsham, Beccles, who claims that it is absolutely silent in handling and free from splashing. The churn is fitted with solid rubber rims at the top and bottom, which act as tyres, and the handles are also covered with rubber. In addition to its welcome quality of silence, the churn is supplied with a lid which fits closely and prevents the contamination which must inevitably occur when the lid is loose and dust and dirt can gain an entrance.

### Another Kind of Blue Book.

Dr. Helen MacMurchy, Chief of the Child Welfare Division in Canada, has issued, through the Canadian Department of Health, new editions of some practical pamphlets, called "The Little Blue-Book." The series contains sixteen booklets dealing with such subjects as "How to Care for the Baby," "Canadians Need Milk," "How to Make Our Canadian Home," "How to Prevent Accidents and Give First Aid." Dr. MacMurchy writes very brightly, and each little book is appropriately illustrated and makes really attractive reading. Published in English and French, the Department of Health issues these booklets free on request, and we imagine that they are much in demand.

### Births and Deaths in Scotland.

A preliminary statement regarding births, marriages and deaths in Scotland during 1923 has been issued by the Scottish Registrar-General. The outstanding facts are a further fall in the birth-rate, while the death-rate is the lowest yet recorded. The infantile mortality rate of the year, 79 per 1,000 births, is, like the general death-rate, the lowest yet recorded. Births registered during the year numbered 111,901, which number is 3,184 less than that of the previous year, 4,030 less than the mean of those of the five preceding years, and 2,666 less than the mean of those of the ten preceding years. Deaths registered during the year numbered 63,284, the smallest number of deaths registered in Scotland in any year since 1861.

### "English Life."

*English Life*, an illustrated monthly, though still in its comparative infancy, is already acquiring a reputation for authoritative articles. Its March number contains contributions from many distinguished people, including Sir William Orpen, the Right Hon. L. S. Amery, Viscount Long, Cardinal Mercier, Mr. Chesterton and Miss Margaret Bondfield. There are also various articles on practical subjects—cookery, motoring, house-building and furnishing—and on these and other matters readers can obtain advice and help, free of charge. In addition a Travel Bureau has been established, and there is also a Puzzle Section presided over by Father Ronald Knox. The headquarters of this excellent publication are at 9 East Harding Street, Fetter Lane, E.C. 4.

### Ill Effects of Cigarette Smoking.

At the inquest held by Dr. Waldo, Coroner for the City of London and Southwark, upon Brigadier-General Sir Hugh Simpson-Blaikie, it was stated that "death was due to fatty degeneration of the heart following chronic disease of the coronary arteries," and that the deceased's habit of " smoking 25 cigarettes a day would be very harmful." Dr. Waldo, in summing up, said that tobacco acted as a direct heart depressant. Apart from the deleterious effect of the nicotine, the combustion of the paper of the cigarette gave off carbon monoxide—one of the most insidious and poisonous gases known. A few cigarettes a day would do no harm, but in excess, when a man was suffering from a fatty heart, with diseased coronary arteries supplying the heart muscles with blood, the results could not be other than harmful.

### The Limbless Hospital.

On its approaching removal from Ducan-road, Shepherd's Bush, the Ministry of Pensions Special Surgical Hospital will form part of Queen Mary's Convalescent Auxiliary Hospitals at Roehampton, or, as it is familiarly called, the Limbless Hospital. New brick huts are being built at Roehampton for the arriving surgical patients. The vacated hospital was an infirmary under the Hammersmith Guardians until it was commandeered in 1916, added to and improved at a cost of £20,000. The change has been much discussed, but Major Picton Phillips, the medical superintendent, is convinced that the 360 men will be no less comfortable at Roehampton.

### A Warning.

The Acting Secretary of the Charity Organisation Society writes to warn secretaries and officials of charities against a woman who has for some time been preying on some of them by a specially adapted form of the confidence trick:— " Stating that her mother (or brother) has left a large sum of money for distribution to charities, she arranges to pay a visit of inspection, and holds out hope of a generous donation. Then, at the moment of departure, she discovers that she has lost her purse, or it may be her return ticket, or both. The Secretary, gratified by the promise of a new hospital ward perhaps, or a chapel, as the case may be—for the lady is magnificent in her undertakings—naturally proffers a loan of anything up to £5, with which the Lady Bountiful disappears. She is described as aged apparently about 45, with high colour and dark eyes, heavily built with round, plump face; height, medium or just above, with the appearance of a well-to-do countrywoman. Her names are legion, and she has given various addresses, usually in Exeter."

### University College Report.

The committee of University College, London, have issued their annual report for the session, 1922-23. The report shows a year of great activity. Students were enrolled from all over Europe, from America, and thirty-six students came from Japan. Adult education was promoted by free lectures, the number of attendances at which has been most encouraging. The new building for anatomy, histology and embryology, provided by the Rockefeller gift for medical education, was opened by the King and Queen last May, and the enlargement and reconstruction of the engineering buildings is now nearly finished, and steps are being taken to provide a fund of £15,000 to complete their equipment. The report includes a summary of the work of two special schools of architecture and librarianship, references to the establishment of a new chemical engineering laboratory, named after the late Sir William Ramsay, and the financial statement for the year, which shows a total expenditure of £167,415 and a fee revenue of £63,826 11s. 6d.

### Further Reduction in Price of Insulin.

The efforts of a great number of scientific and technical workers on the staff of Messrs. Burroughs, Wellcome and Co. have been directed to the solution of the many problems in the production of insulin with the result that great improvement has been effected in the yield and the standard of purity of " Wellcome " brand insulin has been still further raised. Owing to the improved methods of manufacture the firm now announce a further considerable reduction in price. When first issued the cost of a phial of insulin containing 100 units (approximately 10 doses) was 25s., subsequently it was found possible to reduce this price to 17s. 6d., and later increased demand, together with improved methods of production, made it possible further to reduce the price to 12s. 6d. Messrs. Burroughs, Wellcome and Co. have now taken the initiative in a still further reduction, and the 100-unit phial of " Wellcome " brand insulin will henceforth be sold at 6s. 8d. (that is, 8d. per dose of 10 units). Hospitals will be supplied at a still lower rate.

